# DA.*Vra1*-congenic rats display increased gene expression and Schwann cell apoptosis but unaffected nerve regeneration compared to parental DA rats after sciatic nerve injury and repair

**DOI:** 10.3389/fcell.2025.1536347

**Published:** 2025-04-28

**Authors:** Lena Stenberg, Michael Jewett, Alfredo Dueñas Rey, Maria Swanberg, Lars B. Dahlin

**Affiliations:** ^1^ Department of Translational Medicine – Hand Surgery, Lund University, Malmö, Sweden; ^2^ Department of Experimental Medicine, Lund University, Lund, Sweden; ^3^ Department of Hand Surgery, Skåne University Hospital, Malmö, Sweden; ^4^ Department of Biomedical and Clinical Sciences, Linkoping University, Linkoping, Sweden

**Keywords:** nerve injury, nerve regeneration, ATF3, cleaved caspase 3, c-Jun, apoptosis, Hspb1, Vra1

## Abstract

**Introduction:**

The rat *Vra1* locus, containing *glutathione S-transferase alpha 4* (*Gsta4*), regulates the degeneration of central nervous system (CNS) neurons in toxin-, protein-, and injury-based models. We hypothesize that Piebald Virol Glaxo.1AV1 (PVG) alleles in *Vra1* confer protection and increased axonal outgrowth after peripheral nerve injury and repair.

**Methods:**

DA rats (n = 14) and DA rats with PVG alleles in the *Vra1* locus (DA.*Vra1,* n = 14) were subjected to sciatic nerve transection and immediate repair. After 6 days, axonal outgrowth and protein and gene expression were analyzed in injured and uninjured nerves and dorsal root ganglia (DRG).

**Results:**

No differences in axonal outgrowth were observed between strains, but the number of apoptotic Schwann cells in the injured distal nerve end was higher in DA.*Vra1* than in DA rats (p = 0.003). In both strains, gene- and protein expression of activating transcription factor 3 (ATF3) and 27-kDa heat shock protein (HSP27, i.e., Hspb1) were increased in injured vs. uninjured DRG. In DA.*Vra1* rats, *Gsta4* gene expression was lower in injured vs. uninjured DRG (p = 0.043) but higher than in DA rats in injured nerves (p = 0.008) and injured DRG (p = 0.008). DA.*Vra1* had higher gene expression of *Atf3* (p = 0.016) and *caspase 3* (p = 0.032) in injured nerves than DA rats.

**Discussion:**

Results highlight the complexity of nerve injury and repair, supporting further investigation of *Gsta4* in nerve regeneration.

## 1 Introduction

Knowledge about nerve degeneration and regeneration mechanisms after injury, as well as the related variety of involved cell types, including neurons, Schwann cells, and macrophages in nerves and dorsal root ganglia (DRG), is still scarce (Dahlin, 2023; Zigmond and Echevarria, 2019; Balog et al., 2023; Lindborg et al., 2018). Neuronal and Schwann cell survival and cell death, that is, apoptosis, may be one relevant factor behind insufficient outcomes after nerve injury and repair or reconstruction. Schwann cells are crucial for nerve regeneration, where the balance between proliferation and apoptosis regulates some of the involved processes (Dahlin, 2023). Details behind neuronal cell death and apoptosis of Schwann cells in the distal nerve end after nerve injury and repair are still not clarified, and genetic aspects must be highlighted. Naturally occurring allelic variations in the *Vra1* locus on rat chromosome 8 were initially linked to degeneration of motor neurons after ventral root avulsion at the CNS/PNS border (Lidman et al., 2003). Piebald Virol Glaxo.1AV1 (PVG) alleles in *Vra1* (i.e., in DA.*Vra1* congenic rats) conferred a 50% increased survival of motor neurons compared to DA alleles (i.e., in parental DA rats) (Swanberg et al., 2009). DA.*Vra1* congenic rats have also been found to be partially protected from dopaminergic neurodegeneration in toxin- and alpha-synuclein-induced models for Parkinson’s disease (Jewett et al., 2017; Jewett et al., 2018) and from neurodegeneration after traumatic brain injury (Al Nimer et al., 2013). The *Vra1* candidate gene is *glutathione S-transferase alpha 4 (Gsta4*), where PVG alleles confer increased gene expression and neuroprotection (Strom et al., 2012). The Gsta4 protein belongs to the glutathione transferase A4 family of transferases, which are relevant for neurodegeneration and detoxification after brain injury (Jewett et al., 2017; Al Nimer et al., 2013). *Gsta4* gene expression is upregulated in the striatum and midbrain in DA.*Vra1* congenic rats compared to parental DA rats as early as 2 days after a brain lesion at the onset of neurodegeneration (Jewett et al., 2017). Gsta4 is an intrinsic regulator of oligodendrocyte differentiation, survival, and remyelination (Carlstrom et al., 2020). Thus, there is experimental evidence that *Vra1/Gsta4* affects the extent of different types of neurodegenerative and regenerative processes, calling for an investigation of whether *Vra1/Gsta4* also affects peripheral nerve injury and repair processes (Akram et al., 2022; Arthur-Farraj and Coleman, 2021; Lee and Cho, 2021).

Nerve transection and subsequent repair is a common injury in clinical practice, resulting in insufficient outcome (Deumens et al., 2010; Dahlin and Wiberg, 2017), partially related to neuronal cell death in DRG (Welin et al., 2008) and to the timing of surgery (Dahlin and Wiberg, 2017). Substantial molecular changes appear in the nerves and DRG after nerve injury, involving the MAPK pathway with the necessary involvement of c-Jun N-terminal kinase (JNK), activating transcription factor 3 (ATF3), and c-Jun (Jessen and Mirsky, 2016; Lindwall and Kanje, 2005), where ATF3 and c-Jun expressing Schwann cells are balanced during the regeneration process (Stenberg and Dahlin, 2014; Stenberg et al., 2021; Katz et al., 2022). In addition, heat shock protein 27 (also named heat shock protein beta-1; Hspb1), a molecular chaperone, whose expression in the sciatic nerve and DRG is initiated by the MAPK signaling pathway after nerve injury, is neuroprotective (Stenberg et al., 2021; Costigan et al., 1998; Pourhamidi et al., 2011).

The transcription factor nuclear factor (erythroid-derived 2)-like 2 (Nrf2) gene regulates activation through the Nrf2-ARE pathway (David et al., 2017). During normal conditions, Nrf2 activation is low and, as a response to oxidative stress, upregulates in healthy and diabetic *in vitro* and *in vivo* models after nerve injury (David et al., 2017; Ma, 2013). Earlier studies in mice have shown that an absence of transcription factor Nrf2 response can lead to reduced proinflammatory macrophages, thereby delaying nerve regeneration and diminishing functional recovery after a sciatic nerve crush (Kobayashi et al., 2016; Zhang et al., 2013). Interestingly, ATF3 interacts with Nrf2 and regulates its activation in endothelial cells by communication with c-Jun (Kobayashi et al., 2016; Agell et al., 2002). A nerve root avulsion, the most extensive nerve damage a patient may suffer, although less frequently presenting clinically, results in extensive loss of motor neurons and decreased molecular and cellular signals in the spinal cord (Piehl et al., 1999; Koliatsos et al., 1994). In the present study, a milder and clinically more frequently occurring nerve injury model was used to compare the two different strains—DA rats and DA.*Vra1* congenic rats—regarding gene expression and nerve regeneration, particularly axonal outgrowth, evaluated 6 days after a sciatic nerve injury and immediate repair. We hypothesize that PVG alleles in the *Vra1* locus are neuroprotective, with a conceivable effect on axonal outgrowth after a peripheral nerve injury with subsequent nerve repair performed without any delay (Stenberg and Dahlin, 2014; Stenberg et al., 2021).

## 2 Materials and methods

### 2.1 Animals and surgery

Two inbred strains, DA rats and DA.*Vra1* congenic rats, were used in the present study. DA.*Vra1* congenic rats have DA alleles in the background genome and PVG alleles in *Vra1*, a fragment on rat chromosome 8 with 35 genes, including the *Gsta4* gene. The *Vra1* fragment was defined by the D8rat104 and D8Mgh4 markers and transferred from PVG to DA through selective back-crossing for >20 generations. The strains were kindly provided by Professor Fredrik Piehl, Karolinska Institute, Sweden, and bred at the Faculty of Medicine at Lund University, Sweden. The rats were kept under standardized housing conditions with constant room temperature and humidity and with a light-dark cycle of 12 h/12 h. Food and water were provided *ad libitum*.

**FIGURE 1:**
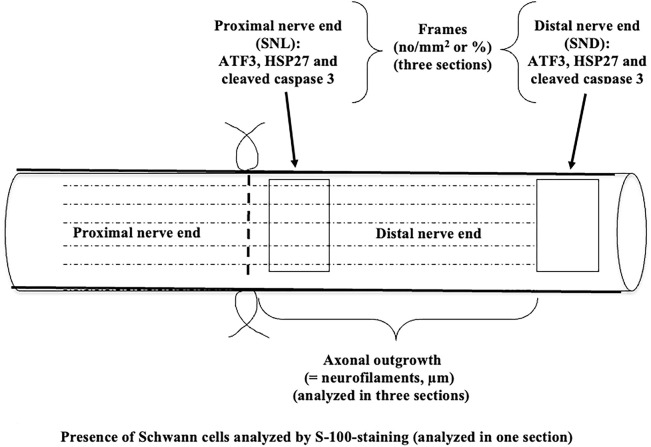
Schematic drawing of the experimental design of nerve injury and immediate repair in DA and DA.*Vra1* congenic strain rats. The specimens were analyzed immunohistochemically 6 days after nerve injury and repair in the proximal (SNL) and in the distal part of the nerve (SND); the latter at a position where the axons had not reached at 6 days. The schematic drawing is modified after Figure 4 from Stenberg and Dahlin (2014).

All experiments were performed in female rats (bodyweight, approximately 200 g) having an age around 30 weeks. Surgery was performed aseptically using general anesthesia with a mixture of Rompun® 20 mg/mL (Bayer Healthcare, Germany) and Ketalar® 10 mg/mL (Pfizer, Finland) at a dose of 0.125 mL/kg bodyweight intraperitoneally. The sciatic nerve was exposed at the hindlimb on the left side in the DA (n = 14) and in the DA.*Vra1* congenic (n = 14) rats, transected, and immediately repaired, without any tension, using 9–0 ethilon sutures at the midthigh level as previously described (Figure 1) (Stenberg and Dahlin, 2014; Yi and Dahlin, 2010; Haastert-Talini et al., 2013; Meyer et al., 2016; Stenberg et al., 2016). Postoperatively, the rats were treated with an intramuscular injection of buprenorphine (0.01–0.05 mg/kg bodyweight; 0.3 mg/mL, Temgesic®, Schering-Plough, Europe, Belgium).

### 2.2 Harvest of specimens and immunohistochemical analyses

At 6 days post-surgery, the repaired sciatic nerves and DRG on the injured side, as well as the corresponding nerve and DRG on the uninjured side, were dissected and harvested. In the immunohistochemical part of the study, nine DA and nine DA.*Vra1* congenic rats were analyzed, while five DA and five DA.*Vra1* rats were used for qPCR analysis. For the immunohistochemistry analyses, both nerves and DRG specimens were processed, and 8-µm-thick sections [at least three sections per sample at different levels in the tissue, except for S-100 staining (one section); Figure 1] were collected on Superfrost® plus glasses (Menzel-Gläser, Germany) using a cryostat as described (Stenberg and Dahlin, 2014; Stenberg et al., 2016). Using the immunohistochemical method, axonal outgrowth was evaluated by staining of neurofilament protein (DAKO, Glostrup, Denmark) in the sciatic nerves, diluted in 1:80 in 0.25% Triton-X 100 (Sigma-Aldrich United States) and 0.25% bovine serum albumin (BSA, Sigma-Aldrich, United States) in phosphate-buffered saline (PBS), and by using the secondary antibody Alexa fluor 594 conjugated goat anti-mouse IgG (Invitrogen, Molecular Probes, United States; dilution 1:500 in PBS).

Activated and apoptotic Schwann cells were stained by rabbit anti-ATF3 polyclonal antibody (1:200; Santa Cruz Biotechnology, United States) and anti-cleaved caspase 3 antibody (1:200; BioNordika, Stockholm, Sweden), both diluted in 0.25% Triton-X 100% and 0.25% BSA in PBS. The secondary antibody, Alexa Fluor 488 conjugated goat anti-rabbit IgG (Invitrogen, Molecular Probes, United States), was used and diluted 1:500 in PBS. The activated or apoptotic Schwann cells were identified by their location and their oval-shaped nuclei, as described earlier (Stenberg and Dahlin, 2014; Tsuda et al., 2011). Double staining with S-100 (S-100 α/β mouse monoclonal IgG) was performed to verify positive ATF3 and cleaved caspase 3 stained Schwann cells in the sciatic nerves. On the first day, sciatic nerves were stained with the primary ATF3 polyclonal or cleaved caspase 3 antibodies. On the second day, following wash with PBS, the slides were incubated with Alexa fluor 488 goat anti-rabbit IgG (Invitrogen, Molecular Probes, USA; diluted 1:500 in PBS) for 2 h in room temperature. The slides were then washed in PBS and incubated with S-100 a/b mouse monoclonal antibody (sc-58839, Santa Cruz Biotechnology Inc., Dallas, TX, USA; diluted 1:300) in 0.25% Triton-X-100 (Sigma-Aldrich, St Louis, MO, USA) and 0.25% bovine serum albumin (BSA; Sigma-Aldrich, St Louis, MO, USA) in PBS overnight at 4°C (Stenberg et al., 2021). On the third and last, the slides were washed again and stained with secondary Alexa fluor 594 goat anti-mouse IgG (Invitrogen, Life Technologies Corporation, Carlsbad, CA, USA; diluted 1:500 in PBS) for 2 h, and finally, all slides were mounted with 4’,6’-diamidino-2-phenylindole (DAPI; VECTASHIELD®, Vector Laboratories Inc, Burlingame, USA) to visualize the cell nuclei (i.e., for calculating total number of cells) and cover slipped.

In separate sections, the expression of HSP27 was measured at the two locations by staining with primary goat anti-HSP27 (sc-1048, Santa Cruz Biotechnology, United States; dilution 1:200 in 0.25% Triton-X-100% and 0.25% BSA in PBS at 4°C). The anti-HSP27 antibody was detected with the secondary Alexa Fluor 488 donkey anti-goat antibody (Molecular Probes, Eugene, Oregon, United States; dilution 1:500) in PBS for 2 h at room temperature, as described by Stenberg et al. (2017).

DRG at L4 and L5 were collected bilaterally and processed as described (Stenberg et al., 2017). Briefly, longitudinal sections (8 µm thickness) were collected on Superfrost® plus glass slides (Menzer-Gläser, Germany), air-dried, washed in PBS, and incubated for ATF3 and HSP27 immunohistochemistry (Stenberg et al., 2017). The secondary antibodies for ATF3 and HSP27 were used as described above for the nerve segments. Sections were coverslipped with VECTASHIELD® (Vector Laboratories, California, United States) containing DAPI for counterstaining of the nuclei for evaluation of activated sensory neurons (i.e., ATF3) and presence of the neuroprotective substance HSP27.

### 2.3 Image analysis

All sections were blind-coded before image analysis. Photos were taken in an Olympus BX3 microscope equipped with a digital camera (Olympus DP80) and analyzed with CellSens Dimension software (Olympus, Tokyo, Japan). Analyses of HSP27 expression in the sciatic nerve and DRG on the images were performed with ImageJ (http://imagej.nih.gov/ij/) as described (Meyer et al., 2016; Hazer Rosberg et al., 2021). The images of neurofilament staining were taken at 10× magnification, and sections of the sciatic nerve and DRG stained for ATF3, cleaved caspase 3, and HSP27 were taken at 10× and 20× (whole DRG 10×) magnification.

In digitalized sections using a Nikon Eclipse fluorescence microscope, the length of the outgrowing axons from the site of suture was measured according to the previous technique (Stenberg and Dahlin, 2014; Whitworth et al., 1996) and expressed as µm. ATF3 and cleaved caspase 3-stained Schwann cells were measured at two different levels in the distal nerve end, that is, immediately distal to the suture line (SNL) and 10 mm from the suture line in the distal nerve end (SND) (Figure 1). The two sites were selected to represent two locations where (a) axons were present together with the Schwann cells (SNL) and (b) in the distal nerve end at a location where the axon has not yet reached according to the length of axonal outgrowth at that specific time point (SND; Figure 1). The image size of these sections was 500 µm × 400 µm. The same squares were used to count the total number of DAPI-stained cells (no/mm^2^). The images were analyzed with NIS-Elements software (Nikon, Kawasaki, Japan), and the ATF3 and cleaved caspase 3-stained Schwann cells were expressed as percentages (Stenberg et al., 2017). HSP27 in the nerve was expressed as previously described (Stenberg et al., 2021).

The number of ATF3-stained sensory neurons was analyzed in the NIS-Elements software (Nikon, Kawasaki, Japan) program as described (Meyer et al., 2016; Stenberg et al., 2017) and expressed as a percentage of total number of sensory neurons. HSP27 expression was stated as a percentage of the total area of the section containing cell bodies; thus, both the intensity of sensory neurons and their satellite cells were included. Finally, the expression of HSP27 in DRG was presented as a ratio between expression at the experimental and control sides.

### 2.4 Gene expression by qPCR

After harvesting the injured (distal to the abovementioned nerve segments) and uninjured sciatic nerves, as well as the corresponding DRG from the DA and DA.*Vra1* rats, the specimens were immediately placed on dry ice. Total RNA was isolated from the distal nerve end (n = 5 randomly selected per strain) and DRG (n = 5 randomly selected per strain) according to former studies (Jewett et al., 2017) through the RNeasy Mini Kit (Qiagen, Hilden, Germany) according to the manufacturer’s instructions steps 4–7. The first three steps were replaced by 600 µm TRIzol (Life Technologies, Warrington, UK) added to each specimen before homogenization with a Fast Prep homogenizer (MP Biomedicals, Burlingame, CA, United States). After the homogenization, all specimens were moved to Eppendorf tubes and resuspended in 0.2 mL chloroform/mL TRIzol, shaken, and centrifuged for 15 min at 12,000 g at 4°. The L4 and L5 DRG from each strain were pooled before the total RNA was converted to cDNA. RNA was quantified by Nanodrop. Preparation of cDNA was performed using the SuperScript™ III First-Strand Synthesis SuperMix (Invitrogen, Waltham, MA, United States) according to the manufacturer’s protocol and thereafter mixed with SsoAdvanced Universal SYBR Green mastermix (BioRad, Hercules, CA, United States). Each PCR well contained 5 µL mastermix, 1 µL nuclease free water, 0.2 µL of each reverse and forward primer, and 3 µL of cDNA. Amplification was performed with 40 cycles (95°C in 15 s, 62°C in 30 s, 68°C for 30 s) and 68°C for 5 min. All samples were run in duplicate or triplicate, and raw data were calculated as the mean value. Expression levels were calculated after normalization against the means of the housekeeping genes. Actb, Rpl13a, and Pgk1 were used as housekeeping genes (Langnaese et al., 2008) (Table 1) and presented as fold change (2^−∆∆Cq^) (Curis et al., 2019). The primers were ordered from Eurofinace (MWG Synthesis Gmbh, Ebersberg, Germany).

**TABLE 1:** List of primers.

Gene	Protein	Forward primer (5′-3′)	Reverse primer (5′-3′)
Atf3	Activating transcription factor 3	GCAGAAGGAGTAGAGAAACTGG	CTGCTTAGCTCTGCAATGTTCC
Jun	Transcription factor Jun	CCAGCAACTTTCCTGACCCA	CTAGCACTCGCCCAACTTCA
Casp3	Caspase 3	GGAGCTTGGAACGCGAAGAA	ACACAAGCCCATTTCAGGGT
Hspb1	Heat shock protein family B (small) member 1 (i.e., HSP27)	TCACCCGGAAATACACGCTC	GGGATGGGTAGCAAGCTGAA
Gsta4	Glutathione S-transferase alpha-4	GACCGTCCTGAAGTTCTAGTGA	TGCCTCTGGAATGCTCTGT
Nrf2	Nuclear factor erythroid 2-related factor 2	CATTTGTAGATGACCATGAGTCGC	TCCTGCCAAACTTGCTCCAT
Actb	Actin, cytoplasmic 1	AAGTCCCTCACCCTCCCAAAAG	AAGCAATGCTGTCACCTTCCC
Rpl13a	Large ribosomal subunit protein uL13	GGATCCCTCCACCCTATGACA	CTGGTACTTCCACCCGACCTC
Pgk1	Phosphoglycerate kinase 1	ATGCAAAGACTGGCCAAGCTAC	AGCCACAGCCTCAGCATATTTC

### 2.5 Statistical analysis

The present nerve injury and repair model is clinically relevant but has the risk of creating surgical variability in results, such as axonal outgrowth. Therefore, the results are presented as median values [25th–75th percentiles] due to the non-normal distribution of data using IBM SPSS Statistics, version 29.0.2.0. The Mann–Whitney test was used to compare the two strains concerning length of axonal outgrowth, numbers of ATF3- and cleaved caspase 3-stained Schwann cells, HSP27 expression in the sciatic nerve, the total number of DAPI cells in the sciatic nerve, numbers and presence of ATF3- and HSP27-stained sensory neurons, and gene expression in the sciatic nerves and DRG. Wilcoxon rank-sum test was used to calculate the differences in ATF3- and HSP27-expression in DRG as well as their gene expression in DRG between the uninjured and injured sides in both strains. Fold change was expressed as mean and 95% confidence interval as an individual ratio from the median value of an applicable reference. A Spearman rank correlation was used to evaluate the associations between the different variables, which were expressed as rho-values and p-values. A linear regression model, adjusted for strain, was used for some of the biomarkers (independent) on axonal outgrowth (dependent). A p-value of less than 0.05 was considered significant.

### 2.6 Ethics

All animal experiments were conducted and approved in accordance with the ethical committee in the Malmö/Lund region in Sweden (permit number M 131/14).

## 3 Results

### 3.1 Axonal outgrowth in the distal nerve end

**FIGURE 2:**
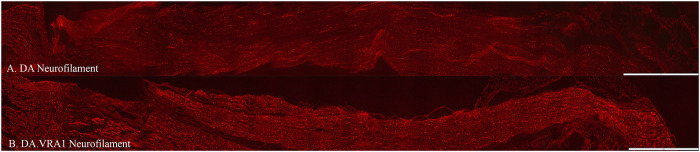
Axonal outgrowth based on neurofilament staining in DA **(A)** and DA.*Vra1* **(B)** rats. Bar = 1,000 µm.

**FIGURE 3:**
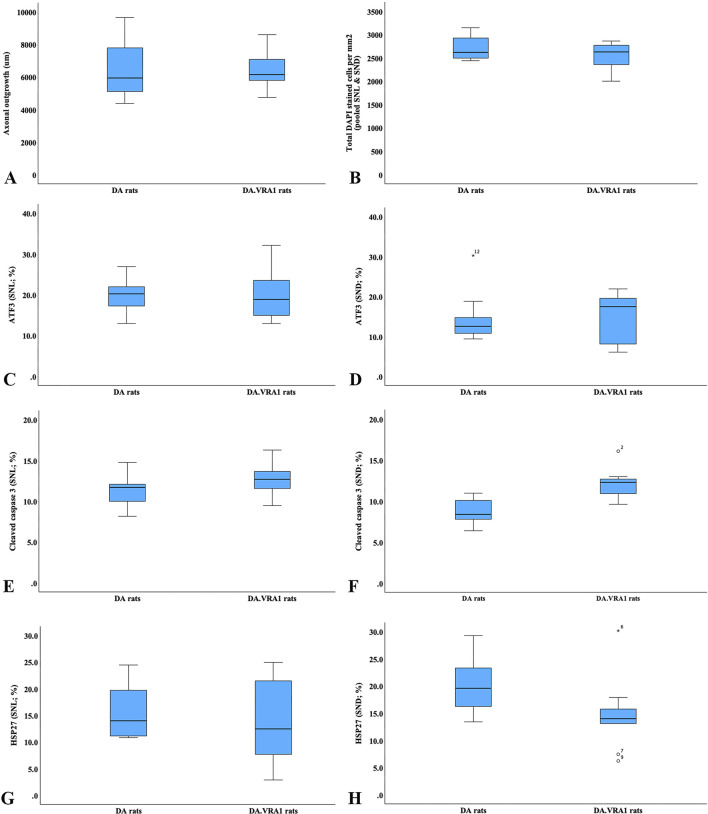
Boxplots of the axonal outgrowth **(A)**, total DAPI-stained cells **(B)**, expression of activating transcription factor 3 (ATF3; **(C, D)**), cleaved caspase 3 **(E, F)**, 27-kDa heat shock protein (HSP27; i.e., Hspb1; **(G, H)**) in DA and DA.*Vra1* rats 6 days after sciatic nerve transection injury and immediate nerve repair. Values are based on the immunohistochemistry of sciatic nerves at the site of the lesion (SNL; **(C, E, G)**) and in the distal nerve ends (SND; **(D, F, H)**). The box plots represent the median (line) with 25th and 75th percentiles (Tukey’s hinge) as well as min–max (outliers are marked as o).

There was no difference between the two strains concerning the length of axonal outgrowth, measured as previously described (Stenberg and Dahlin, 2014; Whitworth et al., 1996), at 6 days (p = 0.93, Table 2; Figures 2, 3A).

**TABLE 2:** Axonal outgrowth and expression of activating transcription factor 3 (ATF3), cleaved caspase 3, and 27-kDa heat shock protein (HSP27; i.e., Hspb1) in DA and DA.*Vra1* rats 6 days after sciatic nerve transection injury and immediate nerve repair. Values are based on the immunohistochemistry of the sciatic nerve at the site of the lesion (SNL) and in the distal nerve end (SND).

	DA (n = 9)	DA*.Vra1* (n = 9)	p-value
Axonal outgrowth (µm)	5,941 [4,879–8,292]	6,166 [5,407–7,380]	0.93
ATF3 (SNL; %)	20.3 [15.7–23.3]	18.9 [14.2–27.8]	0.80
ATF3 (SND; %)	12.6 [10.5–16.9]	17.5 [7.6–20.6]	1.00
Cleaved caspase 3 (SNL; %)	11.7 [9.9–12.9]	12.7 [10.9–13.9]	0.34
Cleaved caspase 3 (SND; %)	8.4 [7.8–10.2]	12.3 [10.5–12.9]	**0.003**
HSP27 (SNL, %)	14.0 [11.0–21.3]	12.5 [6.8–23.0]	0.54
HSP27 (SND, %)	19.6 [15.3–23.4]	14.1 [10.3–16.9]	0.06
Total DAPI-stained cells in pooled SNL and SND (mm^2^)	2,621 [2,490–2,990]	2,633 [2,340–2,801]	0.16

Values are median and 25th–75th percentiles. p-values based on Mann–Whitney U-test, and significant values are marked in bold.

### 3.2 ATF3, cleaved caspase 3, HSP27, and DAPI-stained Schwann cells in the sciatic nerve

**FIGURE 4:**
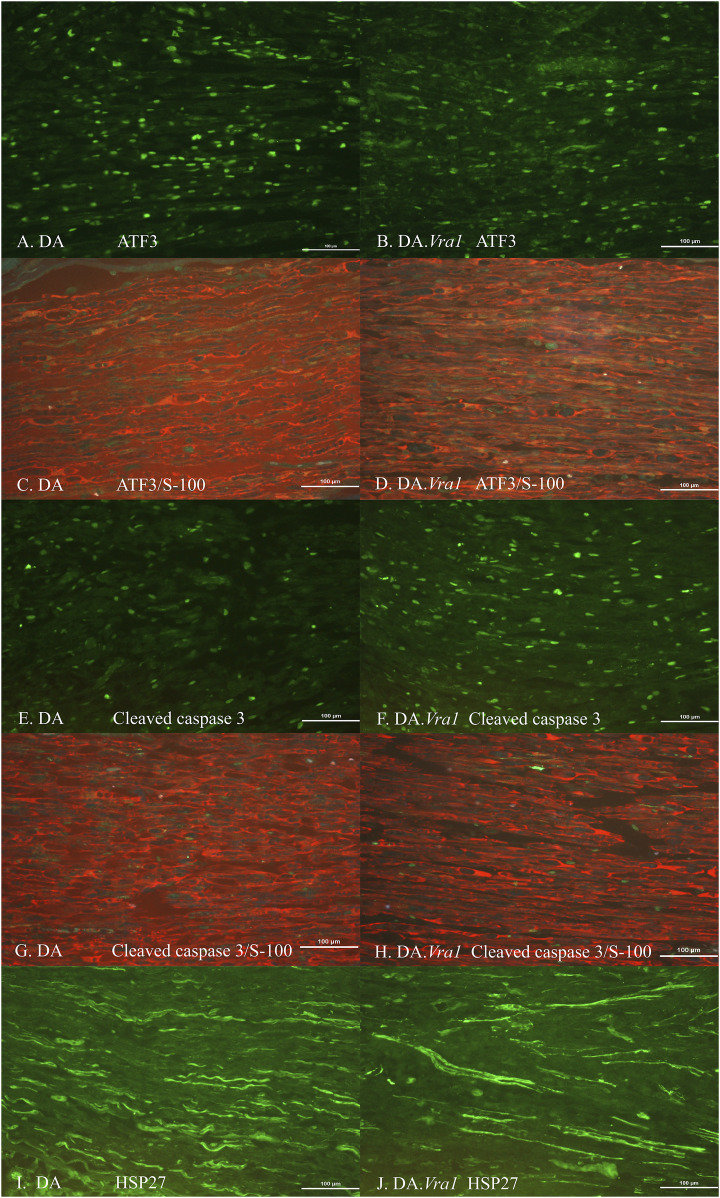
Immunohistochemical staining at the site of the lesion (SNL) of transected and repaired sciatic nerves from DA **(A, C, E, G, I)** and DA.*Vra1* congenic **(B, D, F, H, J)** strain rats, showing staining for ATF3 **(A, B)**, double staining of ATF3/S-100 **(C, D)**, cleaved caspase 3 **(E, F)**, double staining cleaved caspase 3 and S-100 **(G, H)** as well as HSP27 (i.e., Hspb1) **(I, J)**. Bar = 100 µm.

**FIGURE 5:**
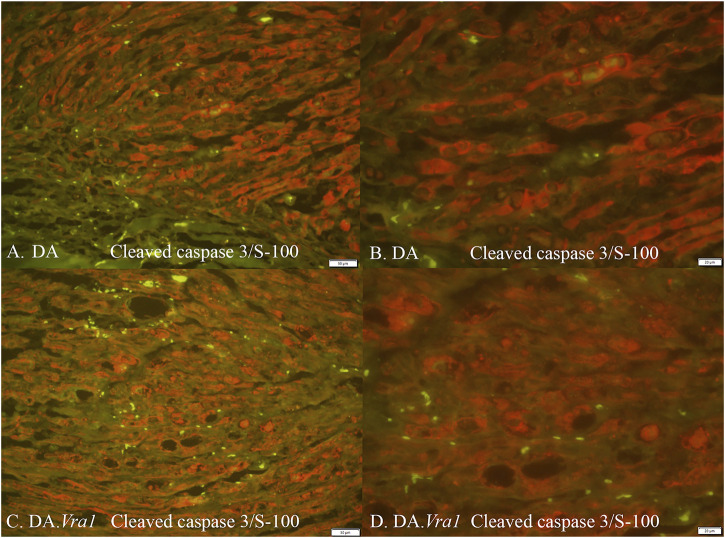
Cleaved caspase 3 and S-100 immunoreactivity (merged) in DA **(A, B)** and DA.*Vra1* **(C, D)** rats evaluated in the distal sciatic nerve (SND) with 20× **(A, C)** and 40× **(B, D)** magnification. Bars in images **(A, C)** = 50 μm and in **(B, D)** = 20 μm.

There were no differences between DA.*Vra1* and DA rats in the number of ATF3-stained Schwann cells immediately distal to the site of the lesion (SNL) (p = 0.80) or in the distal nerve end (SND) (p = 1.00, Table 2; Figures 3C, D; Figures 4A, B). Double staining with the S-100 antibody and ATF3 antibodies, showed that the counted oval formed cells were Schwann cells (Figures 4C, D). Concerning cleaved caspase 3-stained Schwann cells, no difference between the two strains was observed at SNL (p = 0.34), but a higher percentage of cleaved caspase 3-stained Schwann cells was observed in SND in DA.*Vra1* than in DA rats (p = 0.003, Table 2; Figures 3E, F; Figures 4E, F). Double staining with the S-100 antibody and cleaved caspase 3 antibodies showed that the counted oval-formed cells were Schwann cells (Figures 4G, H). Concerning the expression of HSP27 in the sciatic nerve, no differences were found between the two rat strains at SNL (p = 0.54; MW) nor in SND (p = 0.06, Table 2; Figures 3G, H; Figures 4I, J). Merged images, as well as individual channels with higher magnifications of cleaved caspase 3 and S-100 in DA and in DA*Vra1* rats, are shown in Figures 5A–D. Finally, no difference was found in the number of DAPI-stained cells in the distal nerve segment between the two strains (p = 0.16, Table 2, pooled data SNL and SND; Figure 3B).

### 3.3 ATF3 and HSP27 immunoreactivity in sensory neurons in DRG

**FIGURE 6:**
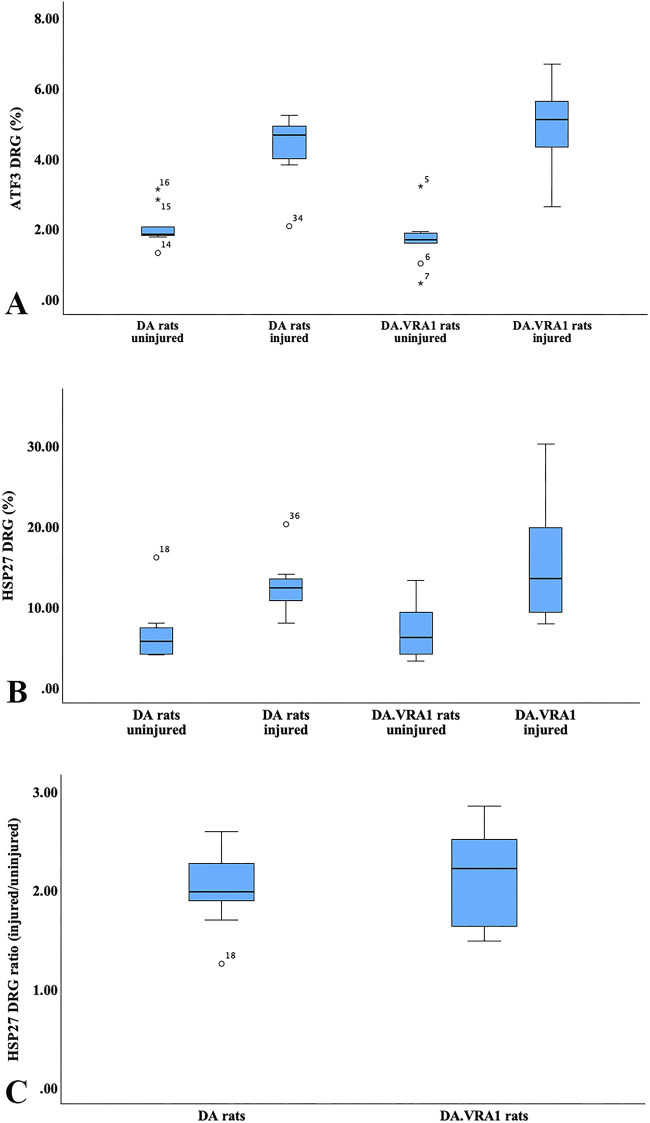
Boxplots of expression of activating transcription factor 3 (ATF3; **(A)**) and 27-kDa heat shock protein (HSP27; i.e., Hspb1; **(B)**) in dorsal root ganglia (DRG) sensory neurons from DA and DA.*Vra1* rats 6 days after a sciatic nerve transection injury and immediate nerve repair. Values are based on the immunohistochemistry of DRG from the uninjured and injured sides. A ratio for HSP27 (injured/uninjured) is presented in **(C)**. The box plots represent the median (line) with 25th and 75th percentiles (Tukey’s hinge) as well as min–max (outliers are marked as o).

**FIGURE 7:**
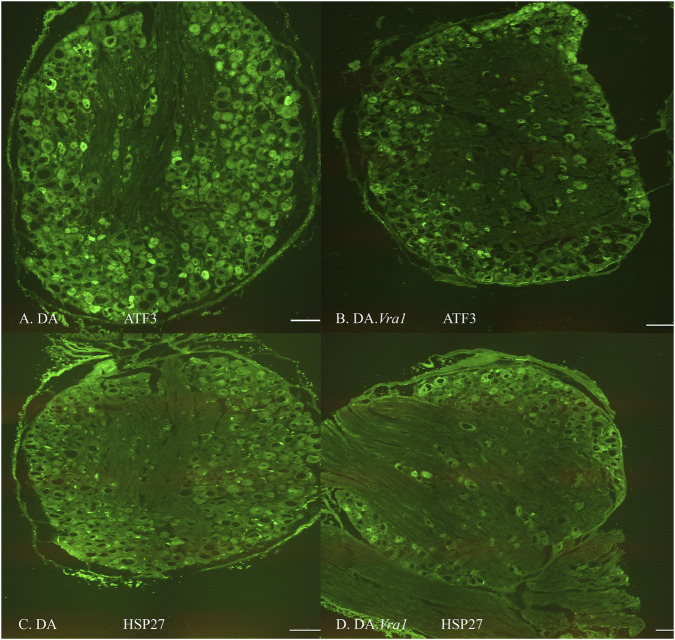
Images of immunohistochemical staining of dorsal root ganglia (DRG) on the injured side where the sciatic nerve was transected and repaired in DA **(A, C)** and DA.*Vra1* congenic **(B, D)** strain rats demonstrating staining for ATF3 **(A, B)** and HSP27 (i.e., Hspb1) **(C, D)**; the latter was analyzed in both sensory neurons and in satellite cells. Bar = 200 µm.

**FIGURE 8:**
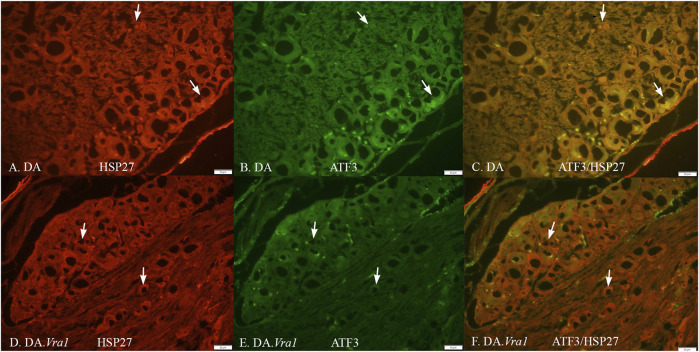
HSP27 **(A, D)** and ATF3 **(B, E)** immunoreactivity in part of a dorsal root ganglion on the injured side, where the sciatic nerve was transected and repaired in DA **(A, B, C)** and DA.*Vra1* **(D, E, F)** rats. Merged images are shown in **(C, F)**. The arrows mark neurons with different sizes in the different images. Bar = 50 μm.

The nerve injury and subsequent repair induced an increased expression of ATF3 and HSP27 in the sensory neurons and satellite cells, respectively, of DRG on the injured side compared to the uninjured side for both rat strains (p = 0.011 and p = 0.008 in DA rats; p = 0.008 and p = 0.008 in DA.*Vra1* rats, Table 3; Figure 6). There were no statistical differences between the strains for ATF3- or HSP27-immunoreactivity in the injured DRG (p = 0.26 and p = 0.73, respectively) or in the uninjured DRG (p = 0.22 and p = 0.86, respectively, Table 3; Figures 6A, B; Figures 7A–D). Double staining of HSP27 and ATF3 in DRG at higher magnification with individual channels and in merged images are shown in Figure 8. The ratio between HSP27 expression in the injured vs. uninjured DRG did not differ between the strains (p = 0.67, Table 3; Figure 6C).

**TABLE 3:** Expression of activating transcription factor 3 (ATF3) and 27-kDa heat shock protein (HSP27; i.e., Hspb1) in dorsal root ganglia (DRG) sensory neurons from DA and DA.*Vra1* rats 6 days after a sciatic nerve transection injury and immediate nerve repair. Values are based on immunohistochemistry of DRG from the uninjured and the injured sides.

	DA (n = 9)	DA*.Vra1* (n = 9)	p-value (DA.*Vra1* vs. DA)
ATF3 uninjured side (%)	1.84 [1.79–2.44]	1.70 [1.30–1.90]	0.22
ATF3 injured side (%)	4.66 [3.90–4.96]	5.11 [3.64–5.79]	0.26
p-value ATF3 injured vs. uninjured	**0.011**	**0.008**	
HSP27 uninjured side (%)	5.69 [4.10–7.66]	6.16 [3.88–10.25]	0.86
HSP27 injured side (%)	12.33 [10.08–13.74]	13.53 [8.72–20.47]	0.73
p-value HSP27 injured vs. uninjured	**0.008**	**0.008**	
HSP27 ratio (injured/uninjured)	1.99 [1.80–2.29]	2.22 [1.62–2.61]	0.67

Values are median and 25th–75th percentiles. p-values are based on the Mann–Whitney U-test (between strains) or the Wilcoxon rank sum test (injured vs. uninjured), and significant values are marked in bold.

### 3.4 Correlation and regression analyses between axonal outgrowth and protein expression

**FIGURE 9:**
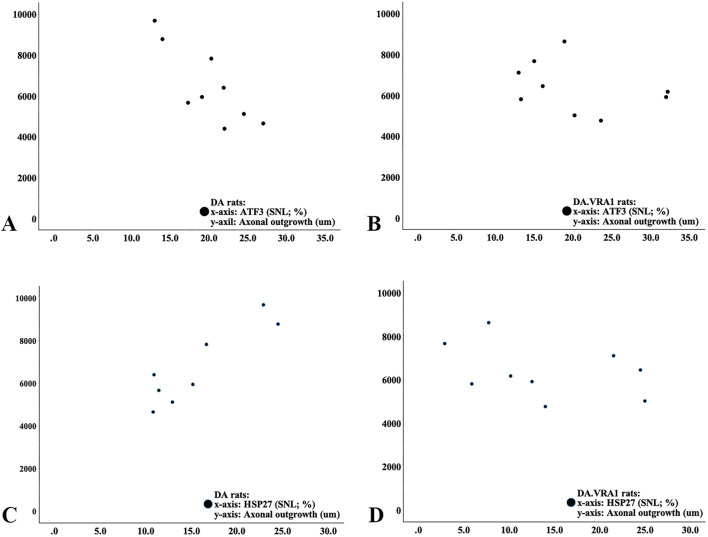
Scatter diagram from correlation analysis in DA rats **(A, C)** and DA.Vra1 rats **(B, D)** showing correlations between ATF3 (%; **(A, B)**) and HSP27 (27-kDa heat shock protein; i.e., Hspb1; %; **(C, D)**) at the proximal part of the nerve (SNL) and axonal outgrowth (µm).

Correlations were investigated between axonal outgrowth and the expression of ATF3, cleaved caspase 3, and HSP27 at the two locations (SNL and SND) in the sciatic nerves as well as for ATF3 and HSP27 in DRG on the injured side. In the DA rats, there was a negative correlation between axonal outgrowth and the number of ATF3-stained Schwann cells at the site of the lesion (rho = −0.80; p = 0.01; Figure 9A) and a positive correlation between the expression of HSP27 at the site of the lesion (rho = 0.81, p = 0.015; Figure 9C) with no other correlations. No correlations between axonal outgrowth and the other biomarkers in either the sciatic nerve or the DRG were found in DA.*Vra1* rats (p > 0.14; Spearman rank correlation test; Figures 9B, D). Furthermore, the linear regression showed a negative association between ATF3 expression at the site of the lesion and axonal outgrowth [−133 (−254 to −12.3; p = 0.033], without any association between the expression of cleaved caspase 3 in the distal nerve end of the sciatic nerve (p = 0.91) or expression of HSP27 ratio in DRG (p = 0.77) with axonal outgrowth (adjusted for strain; which had no association with axonal outgrowth; p = 0.89).

### 3.5 Gene expression in the sciatic nerve

**FIGURE 10:**
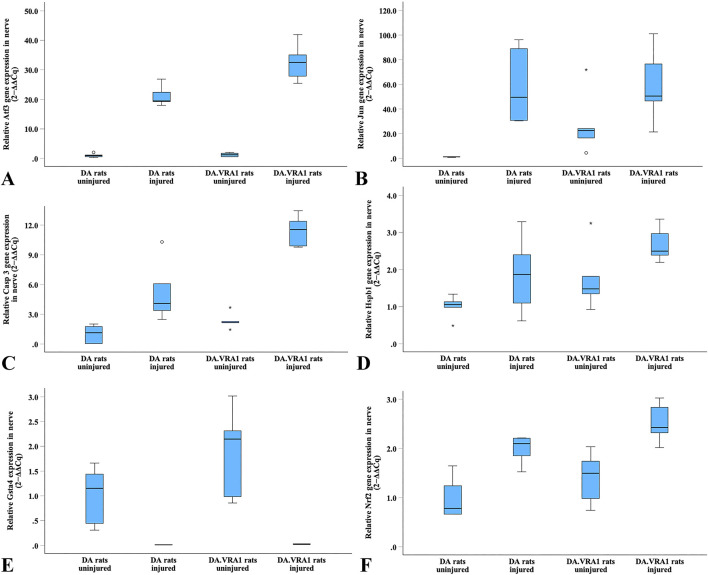
Boxplots of the relative gene expression of *Atf3* **(A)**, *Jun* **(B)**, *Casp 3* **(C)**, *Hspb1* **(D)**, *Gsta4* **(E)** and *Nrf2* **(F)** in the uninjured and injured sciatic nerves in DA and DA.*Vra1* congenic rat strains. The box plots represent the median (line) with 25th and 75th percentiles (Tukey’s hinge) as well as min-max (outliers are marked as o).

Gene expression of *Atf3, Jun, Casp 3, Hspb1,* and *Nrf2* was upregulated in the sciatic nerve after injury in both strains (Figures 10A–D, F). In contrast, *Gsta4* gene expression was dramatically reduced in both strains after injury (Figure 10E). Although very low, DA.*Vra*1 rats had a higher *Gsta4* expression on the injured side than DA rats (p = 0.008, Table 4; Figure 10E).

**TABLE 4:** Gene expression in DA and DA.*Vra1* rats’ uninjured sciatic nerves and sciatic nerves at 6 days after transection and immediate repair (injured). Fold changes were calculated with the DA median as a reference [expressed as mean (95% confidence intervals)].

Gene	Fold change [mean] (95% confidence intervals; CI)]	p-value
DA.*Vra1* uninjured vs. DA uninjured		
*Atf3*	1.25 (0.42–2.09)	0.69
*Jun*	25.13 (−3.70–53.97)	**0.036**
*Casp3*	**2.08 (1.18–2.99)**	**0.032**
*Hspb1*	1.67 (0.62–2.71)	0.10
*Gsta4*	1.62 (0.62–2.61)	0.22
*Nrf2*	1.79 (0.95–2.64)	0.22
DA.*Vra1* injured vs. DA injured		
*Atf3*	**1.68 (1.25–2.11)**	**0.016**
*Jun*	1.20 (0.42–1.97)	1.00
*Casp3*	**2.79 (2.31–3.27)**	**0.032**
*Hspb1*	1.43 (1.12–1.74)	0.22
*Gsta4*	**1.94 (1.36–2.52)**	**0.008**
*Nrf2*	1.20 (0.96–1.44)	0.06

p-values are based on the Mann–Whitney U-test; p-values <0.05 in bold.

*Casp3* had a higher expression in DA.*Vra1* congenic rats than parental DA rats at both the injured and uninjured side (p = 0.032 for both, Table 4; Figure 10C). Gene expression was higher in DA.*Vra1* than DA rats on the injured side for *Atf3* (p = 0.016, Table 4; Figure 10A) and on the uninjured side for *Jun* (p = 0.036, but 95% CI spanning from −3.70 to 53.97, Table 4; Figure 10B). *Hspb1* and *Nrf2* gene expressions did not differ between DA.*Vra1* and DA rats (Table 4; Figures 10D, F).

### 3.6 Gene expression in DRG

**FIGURE 11:**
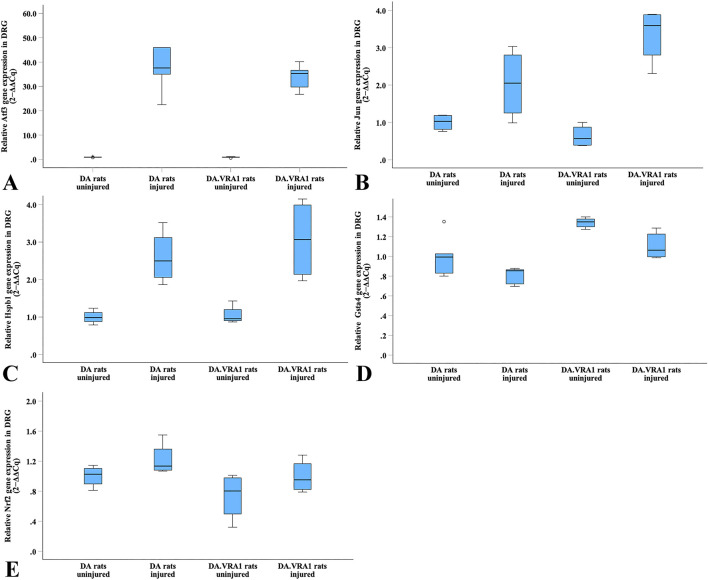
Boxplots of the relative gene expression of *Atf3* **(A)**, *Jun* **(B)**, *Hspb1* **(C)**, *Gsta4* **(D)** and *Nrf2* **(E)** in DRG on the uninjured and injured sides in DA and DA.*Vra1* congenic rat strains. The box plots represent the median (line) with 25th and 75th percentiles (Tukey’s hinge) as well as min–max (outliers–marked as o).

*Atf3* gene expression was significantly upregulated in DRG after injury in both strains (p = 0.043 for both strains, Table 5; Figure 11A), with no difference between the strains. Gene expression levels of *Jun, Hspb1*, and *Nrf2* were not significantly altered in DRG after injury, nor were there significant differences between the strains (Table 5; Figures 11B, C, E).

**TABLE 5:** Gene expression in DA and DA.*Vra1* rats’ uninjured dorsal root ganglia (DRG) and DRG 6 days after sciatic nerve transection and immediate repair. Fold changes were calculated with uninjured as a reference for within-strain comparisons and with DA as a reference for between-strain comparisons. [Fold change expressed as mean (95% confidence intervals)].

Gene of interest	Fold change [mean] (95% confidence intervals; CI)	p-value
DA injured vs. DA uninjured		
*Atf3*	**43.3 (23.2–63.3)**	**0.043**
*Jun*	1.98 (0.52–3.44)	0.07
*Hspb1*	2.62 (1.47–3.76)	0.07
*Gsta4*	0.81 (0.70–0.92)	0.08
*Nrf2*	1.19 (0.85–1.54)	0.11
DA.*Vra1* injured vs. DA.*Vra1* uninjured		
*Atf3*	**34.6 (27.6–41.5)**	**0.043**
*Jun*	5.91 (3.82–7.99)	0.07
*Hspb1*	3.23 (1.40–5.05)	0.07
*Gsta4*	**0.82 (0.70–0.95)**	**0.043**
*Nrf2*	1.24 (0.79–1.68)	0.07
DA.*Vra1* uninjured vs. DA uninjured		
*Atf3*	1.00 (0.68–1.31)	1.00
*Jun*	0.61 (0.15–1.07)	0.11
*Hspb1*	1.06 (0.65–1.47)	0.89
*Gsta4*	1.35 (1.29–1.42)	0.06
*Nrf2*	0.72 (0.23–1.21)	0.20
DA.*Vra1* injured vs. DA injured		
*Atf3*	0.90 (0.72–1.08)	0.55
*Jun*	1.63 (1.06–2.21)	0.11
*Hspb1*	1.26 (0.55–1.97)	0.49
*Gsta4*	**1.30 (1.10–1.49)**	**0.008**
*Nrf2*	0.88 (0.56–1.19)	0.20

p-values are based on the Mann–Whitney U-test; p-values <0.05 in bold.

Within the DA.*Vra1* strain, *Gsta4* gene expression was significantly lower in DRG on the injured side than on the uninjured side (p = 0.043, Table 5; Figure 11D). As expected from previous findings, *Gsta4* gene expression was higher in DA.*Vra1* rats than DA rats (injured side p = 0.008, uninjured side p = 0.06, Table 5; Figure 11D).

## 4 Discussion

The extent of nerve regeneration processes can be investigated using a short-term peripheral nerve injury and repair model, with analyses of different biomarkers related to peripheral nerve regeneration. This model is in accordance with other models used to investigate the impact of HSP27 on axonal outgrowth after nerve injury and repair (Stenberg et al., 2021) and the influence of the lactoferrin-derived peptide PLX01 (Hazer Rosberg et al., 2024). DA.*Vra1*-congenic strain rats and their parental DA rats are relevant models to study the genetic regulation of nerve injury because previous studies have found a protective effect of the *Vra1* locus, with less neurodegeneration after a severe injury, like ventral root avulsion at the spinal CNS/PNS border (Swanberg et al., 2009), after a traumatic brain injury (Al Nimer et al., 2013), and in toxin- or alpha-synuclein-induced models for Parkinson’s disease (Jewett et al., 2017; Jewett et al., 2018). The neuroprotective effect is thought to be mediated by *Gsta4*, where the PVG alleles in DA.*Vra1* rats confer an increased gene expression (Strom et al., 2012). In this study, we compared outcomes in DA.*Vra1* and DA rats 6 days after sciatic nerve injury and repair using axonal outgrowth as one primary endpoint.

During normal conditions after a peripheral nerve injury and repair in rats, Schwann cells are dynamic and able to change phenotypes from a no-repair to a repair state, with concurrent changes in the expression of genes and protein activation (Jessen and Mirsky, 2016). The injury-associated protein cleaved caspase 3 is generally observed after nerve injury and repair in the sciatic nerve (Dahlin, 2023; Stenberg and Dahlin, 2014; Hazer Rosberg et al., 2021). We found a higher number of cleaved caspase 3-immunostained Schwann cells in the distal part of the nerve (SND), a location that axons had not reached during the regeneration process, but not close to the nerve repair (SNL; axons present) in DA.*Vra1* compared to DA rats. The differences in gene expressions in the sciatic nerve and DRG between the two strains were limited.

However, *Atf3* and *Gsta4* showed increased fold change in the injured sciatic nerve in the DA.*Vra*1 rats compared to DA rats. ATF3 protein increases the intrinsic state of growth in sensory neurons in DRG (Seijffers et al., 2007; Saito and Dahlin, 2008) and may be related to an improved axonal outgrowth. GSTA4 protein has a neuroprotective function in the CNS (Lidman et al., 2003; Swanberg et al., 2009; Jewett et al., 2017; Jewett et al., 2018; Al Nimer et al., 2013; Strom et al., 2012) and may regulate differentiation, survival, and remyelination of oligodendrocytes (Carlstrom et al., 2020), but the potential effects on Schwann cells or peripheral nerve regeneration are not known. Despite the potential impact of *Atf3* and *Gsta4* on nerve regeneration, no difference in the length of axonal outgrowth was observed (i.e., no difference between strains). In contrast, DA.*Vra1* rats had a higher *Casp3* gene expression in both the injured and uninjured sciatic nerves than DA rats. This suggests that the *Vra1* locus with increased *Gsta4* gene expression leads to the enhanced Schwann cell apoptosis that was observed by the immunohistochemical staining. This could, in turn, counteract any stimulatory effect of *Atf3* in axonal outgrowth in the sciatic nerve. The corresponding comparison of gene expressions in DRG showed only an increased fold change in *Gsta4* in DA.*Vra1* rats. These alterations, together with the non-significant fold change difference of *Hspb1* (i.e., *HSP27*) between DA.*Vra1* and DA rats, may not be sufficient to induce any increased axonal outgrowth after nerve injury and repair.

HSP27 is a neuroprotective chaperone and seems not to be related to axonal outgrowth (Stenberg et al., 2021). The lack of effects on axonal outgrowth could be due to the increased number of cleaved caspase 3-stained Schwann cells in the distal nerve end (SND, at the location where outgrowing axons have not reached) that potentially impacts the nerve regeneration process. Consequently, when outgrowing axons reach an area of Schwann cells in the injured nerve, the number of cleaved caspase 3-stained Schwann cells is “normalized” between DA.*Vra1* and DA rats (i.e., at SNL). These findings indicate the intricate balance and complex interaction between the outgrowing axons and the Schwann cells after nerve injury and repair or reconstruction.

ATF3 and HSP27 are biomarkers that represent activated and neuroprotected Schwann cells and sensory neurons, respectively (Stenberg et al., 2021; Stenberg et al., 2016; Hazer Rosberg et al., 2021). The expression of these and other proteins, like PACAP, may vary depending on size of the sensory neurons (small-, medium-, or large-sized or diameter of neurons > or <35 µm) as well as on type of nerve injury, being different from the currently used injury and repair method (Saito and Dahlin, 2008; Shortland et al., 2006; Cooper et al., 2024; Hunt et al., 2012; Pettersson et al., 2004). The pattern of up- and downregulated levels of different proteins as a response to injury is complex and depends on the severity of the injury and the time after injury, where the present model represents a short-time evaluation model (Hunt et al., 2012; Pettersson et al., 2004; Oblinger et al., 1989; Wong and Oblinger, 1987; Delibaş et al., 2024).

The expressions of ATF3 and HSP27 were not evaluated in different sensory neurons based on size because a primary aim was to investigate any difference in axonal outgrowth between the strains. ATF3- and HSP27-immunostainings of sensory neurons and satellite cells in DRG were increased after nerve injury and repair in both strains. It is not possible to quantify satellite cells using the listed imaging modalities, but such cells respond to injuries as well (Delibaş et al., 2024). Surprisingly, there was a negative association between axonal outgrowth and ATF3-stained Schwann cells at the sites of lesions adjusted for strain (SNLs), which contrasts with previous data from the same injury model in Wistar rats (Stenberg and Dahlin, 2014). We did not observe any strain differences in ATF3- or HSP27-stained Schwann cells in the distal sciatic nerve end (SND), at the site of injury and repair (SNL) or in the DRG.

A recent study found no association between HSP27 expression and the occurrence of nerve entrapment disorders, which are milder forms of nerve injury, such as carpal syndrome and ulnar nerve entrapment in humans (Bergsten et al., 2022), indicating that different nerve injury models are needed to elucidate the complex pattern of gene- and protein expression. In line with the findings of ATF3- and HSP27 expression, we found no difference on axonal outgrowth between the two strains in this short-time model. The length of outgrowing axons, detected by neurofilament staining on three sections from the nerve at different levels, was measured in accordance with a previously used technique (Stenberg and Dahlin, 2014; Whitworth et al., 1996; Whitworth et al., 1995) and is not expected to differ from a method where the immunohistochemically stained area of outgrowing axons was evaluated despite other used antibodies; a technique that gives similar results (Whitworth et al., 1996; Whitworth et al., 1995). Thus, we cannot expect that another method than the presently used would yield different results.

ATF3 is regulated by the transcription factor c-Jun, which is important for nerve regeneration processes, such as cell survival and proliferation in the sciatic nerve and DRG (Lindwall et al., 2004; Lonze and Ginty, 2002; Blom et al., 2014). *In vitro* and *in vivo* studies have shown an intimate necessity of c-Jun for the upregulation of ATF3 in nerves and in DRG neurons (Lindwall et al., 2004; Raivich et al., 2004). One of the main functions of c-Jun is to regulate the Schwann cell repair program after nerve injury, including the different characteristics of the Schwann cells required for successful nerve regeneration (Jessen and Mirsky, 2016). In the present study, gene expression was higher in DA.*Vra1* than DA rats in the uninjured sciatic nerve for *Jun* and in the injured sciatic nerve for *Atf3*, indicating a higher capacity for nerve regeneration after injury and repair in DA.*Vra1* rats. However, there were no *Jun* gene expression differences between the strains in uninjured or injured DRG, again indicating the complexity of nerve regeneration and the need for evaluation of nerve regeneration in different models (Hazer Rosberg et al., 2024).

*Gsta4* gene expression was lower in the injured than uninjured DRG in both DA.*Vra1* and DA rats, although it was slightly higher in the injured DRG in DA.*Vra1* than DA rats. This is in accordance with data from ventral root avulsion and models for Parkinson’s disease (Swanberg et al., 2009; Jewett et al., 2017; Jewett et al., 2018). Even if the gene expression data is in accordance, there is a discrepancy between the present findings and previous publications related to the investigated neuronal populations, that is, sensory and motor neurons, respectively. However, the location of *Gsta4* transcripts, whether in sensory or motor neurons, Schwann cells, or inflammatory cells, was not presently investigated by immunohistochemistry. A natural variation in *Gsta4* expression in rats that affects degeneration after a traumatic brain injury has also been described (Al Nimer et al., 2013) but is not narrated concerning a peripheral nerve injury. Motor and sensory neurons may behave differently after nerve injury and repair and reconstruction, where misdirection of axonal outgrowth is more relevant among the sensory neurons concerning outcome and functional recovery.

No statistical differences were observed in the *Nrf2* gene expression after nerve injury and immediate repair in either the DA.*Vra1* strain or in the parental DA strain rats. The NRF2 factor acts through the NRF2-ARE signaling pathway, which is a relevant mechanism for defense against oxidative stress and is reported to play an important role in inflammation, management of neuropathic pain, the occurrence of peripheral neuropathy, and after a peripheral nerve injury. In mice with a nerve crush inducing nerve degeneration and regeneration (Zhang et al., 2013; Szepanowski et al., 2017), there is an indication of a connection between delayed nerve regeneration, the efficiency of macrophage accumulation, and a lack of NRF2 (Zhang et al., 2013). Based on the present study, there is no indication that gene expression of *Nrf2* is affected by nerve injury or by the *Vra1* locus in the present rat models, which is why nuclear translocation of NRF2 was not investigated, in addition to the lack of difference in axonal outgrowth between strains.

The mentioned connection between macrophages and NRF2 is worth a judgment. An inflammatory response, with an evaluation of proinflammatory and pro-healing macrophages (Hazer Rosberg et al., 2024), is one mechanism involved in nerve degeneration and regeneration. The *Vra1* locus may be specifically connected to the neurodegeneration of neurons after specific disorders and not to the degeneration process in the nerve trunk, which may be more relevant in other nerve injuries. Because no differences were noted in *Nrf2* or in axonal outgrowth between the strains, the latter being one primary aim, analysis of the inflammatory response was not investigated.

We conclude that genetic models are valuable to further understand the nerve regeneration process after various injuries and repairs or reconstructions. Despite the lack of differences in axonal outgrowth between DA.*Vra1* congenic and DA rats, we found injury- and *Vra1*-regulated expression of several proteins and genes relevant for nerve regeneration, as well as of apoptotic Schwann cells in the distal sciatic nerve end after injury and repair.

## Data Availability

The datasets analyzed in this study can be found in the Database “Geneexpr Stenberg et al Nov 26 2024” and in “Database Immuno Stenberg et al Nov 26 2024”.
